# Detection of EpCAM-positive microparticles in pleural fluid: A new approach to mini-invasively identify patients with malignant pleural effusions

**DOI:** 10.18632/oncotarget.6581

**Published:** 2015-12-12

**Authors:** Elisa Roca, Romaric Lacroix, Coralie Judicone, Sophie Laroumagne, Stéphane Robert, Sylvie Cointe, Alexandre Muller, Elise Kaspi, Patrice Roll, Alain R. Brisson, Claudio Tantucci, Philippe Astoul, Françoise Dignat-George

**Affiliations:** ^1^ VRCM, UMR-S1076, Aix-Marseille Université, INSERM, Faculté de Pharmacie, Marseille, France; ^2^ Division of Thoracic Oncology, Pleural Diseases, and Interventional Pulmonology, Hôpital Nord, AP-HM, Marseille, France; ^3^ Cattedra di Malattie dell'Apparato Respiratorio, Università degli Studi di Brescia, Brescia, Italia; ^4^ Hematology and Vascular Biology Department, CHU La Conception, APHM, Marseille, France; ^5^ R and T department, BioCytex, Marseille, France; ^6^ AP-HM, Hôpital de la Timone, Service de Biologie Cellulaire, Marseille, France; ^7^ Aix-Marseille Université, INSERM, GMGF UMR_S910, Marseille, France; ^8^ UMR-CBMN University of Bordeaux-CNRS-IPB, Pessac, France; ^9^ Aix-Marseille Université, Marseille, France

**Keywords:** extracellular vesicles, microparticles, pleural effusion, pleural neoplasia, EpCAM

## Abstract

Pleural biomarkers allowing to mini-invasively discriminate benign from malignant pleural effusions are needed. Among potential candidates, microparticles (MPs) are extracellular vesicles that vectorize antigen derived from the parent cell. We hypothesized that tumor-derived MPs could be present in the pleural liquid and help to identify patients with malignant pleural effusions. Using highly sensitive flow cytometry and cryo-electron microscopy, we showed that large amounts of MPs from hematopoïetic and vascular origin could be detectable in pleural fluids. Their level did not differ between benign (*n* = 14) and malignant (*n* = 71) pleural effusions. Analysis of selected tumoral associated antigens (podoplanin, mucin 1 and EpCAM, epithelial-cell-adhesion-molecule) evidenced for the first time the presence of tumor-derived MPs expressing EpCAM in malignant pleural fluids only (Specificity = 93%, Sensitivity = 49% and 45% for flow cytometry and ELISA, respectively). The detection of EpCAM-positive-MPs (EpCAM + MPs) by flow cytometry showed a better specificity and sensitivity than ELISA to distinguish between pleural carcinoma and the others malignant pleural effusions (MPE; Sp: 96% *vs* 89%; Se: 79% *vs* 66%). Combining EpCAM+ MPs and cytology improved the diagnosis of MPE compared to cytology alone. This study establishes the basis for using EpCAM+ MPs as a promising new biomarker that could be added to the armamentarium to mini-invasively identify patients with malignant pleural effusions.

## INTRODUCTION

Malignant pleural effusion (MPE) is very common in cancer patients [1, 2] reflecting the dissemination of malignancy as well as advanced disease [3]. The differential diagnosis is based on invasive approaches such as thoracoscopy which has a high diagnostic yield and represents the gold-standard at the present time. Pleural biomarkers which could discriminate benign and malignant pleural effusion are needed for the diagnosis but also for monitoring during the patient's follow-up. Among potential candidate biomarkers, microparticles (MPs) are Extracellular Vesicles (EVs) released by all eukaryotic cells including cancer cells [4]. MPs result from the blebbing of cell membranes in response to activation or apoptosis and vectorize antigens from their parent cells. MPs are characterized by size, ranging from 0.1 and 1 micron. They generally express the anionic phospholipid phosphatidylserine (PS) and membrane antigens representative of their parental cells [5]. These characteristics distinguish MPs from exosomes which are smaller in size, devoid of PS and originate from multivesicular bodies.

The presence of MPs and exosomes has been reported in human body fluids including plasma and other liquids such as bronchoalveolar liquid, urine and ascites fluid [6–9]. To our knowledge, little is known about the presence of MPs in pleural fluid [10, 11]. Because tumor cells produce high numbers of MPs [12], we hypothesized that tumor-derived MPs could be present in pleural fluid and help to mini-invasively discriminate benign from MPE.

## RESULTS

**Figure 1:**
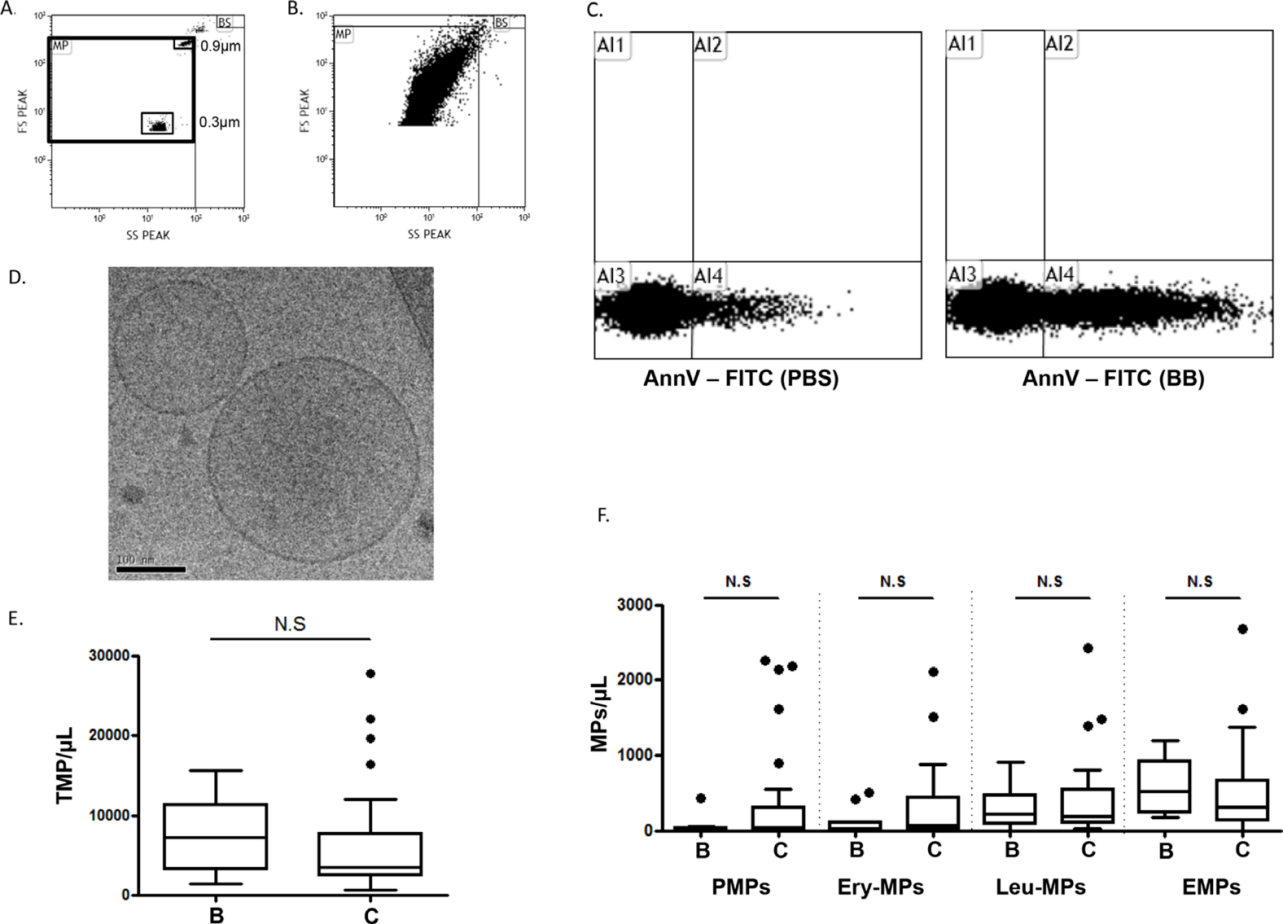
Microparticles in pleural effusions **A.** Flow cytometry scattergram of the microparticle (MP) window of analysis determined by FSC-Megamix-Plus beads. **B.** Representative scattergram of the pleural fluid events appearing in the MP window. **C.** Representative dot plot showing the annexin-V (AnnV) positivity of the pleural fluid extracellular vesicles. The control experiment was performed in the presence of phosphate buffered saline buffer (PBS) compared to Ca2+-containing binding buffer (BB). **D.** Representative image of pleural fluid extracellular vesicles by cryo-transmission electron microscopy. **E.** Total MP counts by flow cytometry (TMP = AnnV+MPs) between benign B. and cancer C. pleural fluids. **F.** Hematopoietic and vascular MP subpopulation enumeration by flow cytometry between benign B. and cancer C. pleural effusions. Platelet-derived MPs (PMPs): AnnV+/CD41+; erythrocyte-derived MPs (Ery-MPs): AnnV+/CD235a+; Leucocyte-derived MPs (Leu-MPs): AnnV+/CD11b+; endothelial-derived MPs (EMPs): AnnV+/CD41−/CD31+. NS = no significant difference.

We investigated the presence of MPs in pleural fluids using highly sensitive flow cytometry. As shown in Figure 1, extracellular vesicles with light scatter properties compatible with those of MPs (Figure 1A and 1B) and positive for Ann-V+ (Figure 1C) were detectable in pleural fluids of neoplastic and non-neoplastic etiologies (*n* = 85). Additionally, Cryo-Transmission Electron Microscopy analysis (cryo-TEM; Figure 1D) confirmed the presence of extracellular vesicles with sizes ranging from 0.1 and 0.5 μm. These features were compatible with MP definition. Using Ann-V labeling, no significant differences in the total MP count were found between benign and MPE (3500 MPs/μL [2400–7800] *vs* 7300 MPs/μL [3200–11000], respectively; *p* = 0.18) (Figure 1E). To characterize the cellular origin of these MPs, we first performed complementary immunophenotyping with antibodies specific for erythrocyte- (EryMP), platelet- (PMP), leukocyte- (LMP) and endothelial-derived MPs (EMP). As illustrated in Figure 1F, the level of MPs from hematopoïetic and vascular origin which does not differ between benign and MPE. We concluded that MPs from hematopoietic and vascular origins failed to discriminate benign from malignant pleural effusions. These results are in agreement with the notions that inflammation and vascular activation are common features of pleurisies regardless of origin.

**Figure 2:**
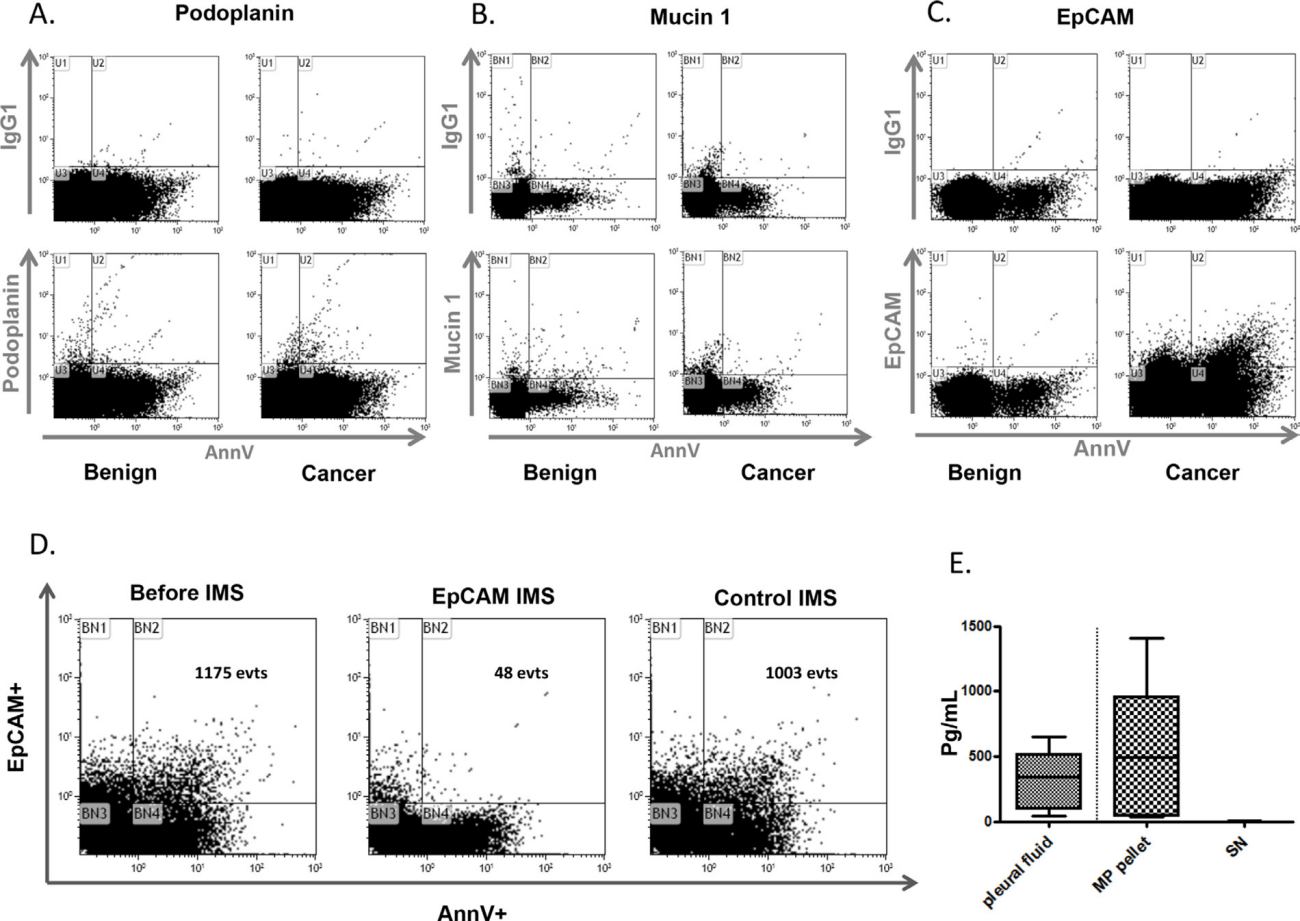
Tumoral microparticles in pleural effusion Representative flow cytometry graphs of podoplanin **A.** mucin 1 **B.** and EpCAM **C.** labeling on MPs from benign B. or cancer C. pleural fluids. The control experiments with appropriate isotype antibodies are displayed above each specific graph. **D.** The specificity of EpCAM+ microparticles in malignant pleural effusions. Representative experiment of AnnV+/EpCAM+MP labeling by flow cytometry before and after immunomagnetic separation (IMS) using beads coated with the EpCAM antibody. The control IMS was performed with beads coated with an irrelevant antibody. **E.** The EpCAM antigens are vectorized by MPs. Comparison of the EpCAM antigen determined by ELISA between the pleural fluids, MP pellets and last-wash supernatants (SN) (*n* = 5).

In both malignant and benign pleural effusions, the detection of about 70% of the Ann-V+MPs (4480+/− 4830 MPs/μL) that fail to express vascular or hematopoietic markers, prompted us to investigate the presence of selected tumor-associated markers. MPE can be divided into primary pleural cancer (malignant pleural mesothelioma) or secondary pleural metastases from other neoplasia (lung, breast, prostate...). Among metastatic pleural cancers, lung cancer is the most frequent etiology. Therefore, we analyzed the most common immunohistochemical markers used in the differential diagnosis between epithelioid pleural mesothelioma and lung adenocarcinoma [17]. Among them, we choose surface markers which are present at the cell membrane and therefore potentially present at the MP surface : podoplanin, mucin 1 and EpCAM. Podoplanin+MPs and mucin 1+MPs were found in pleural effusions of both cancer and benign origin (Figure 2A and 2B). Therefore both podoplanin and mucin 1+ failed to assign the malignant etiology of pleural fluid. This is consistent with the expression pattern of podoplanin found to be upregulated in mesothelioma and other human cancers [18, 19]. However, podoplanin is also expressed in mesothelial cells and other normal tissues [20, 21]. Similarly, mucin 1 can be expressed in tumoral and normal tissues including lung, mammary gland, uterus, esophagus, stomach, duodenum, pancreas, prostate, and hematopoietic cells [22, 23].

By contrast, significant amounts of AnnV+ EpCAM+ events were detected in malignant pleural effusions only (Figure 2C). To investigate whether these events could be specific for EpCAM, an immunomagnetic depletion (IMS) was performed using beads coated with an anti-EpCAM antibody. After IMS, more than 90% of the Ann-V+/EpCAM+ MPs were removed (Figure 2D) whereas no depletion was observed when IMS was performed with beads coated with an irrelevant antibody. These results demonstrate that flow cytometry can be used to specifically detect EpCAM+ MPs in MPE. Moreover, in order to compare the proportion of EpCAM bound to MPs or released as a soluble form, we used high speed centrifugation to separate the vesicular from the soluble fractions. Interestingly, as illustrated in Figure 2E, we found that most of EpCAM antigen was detectable in the pellet whereas the amount remaining in the supernatant was non significant. These results demonstrate that the majority of EpCAM detectable in pleural fluid is bound on the MP surface.

**Figure 3:**
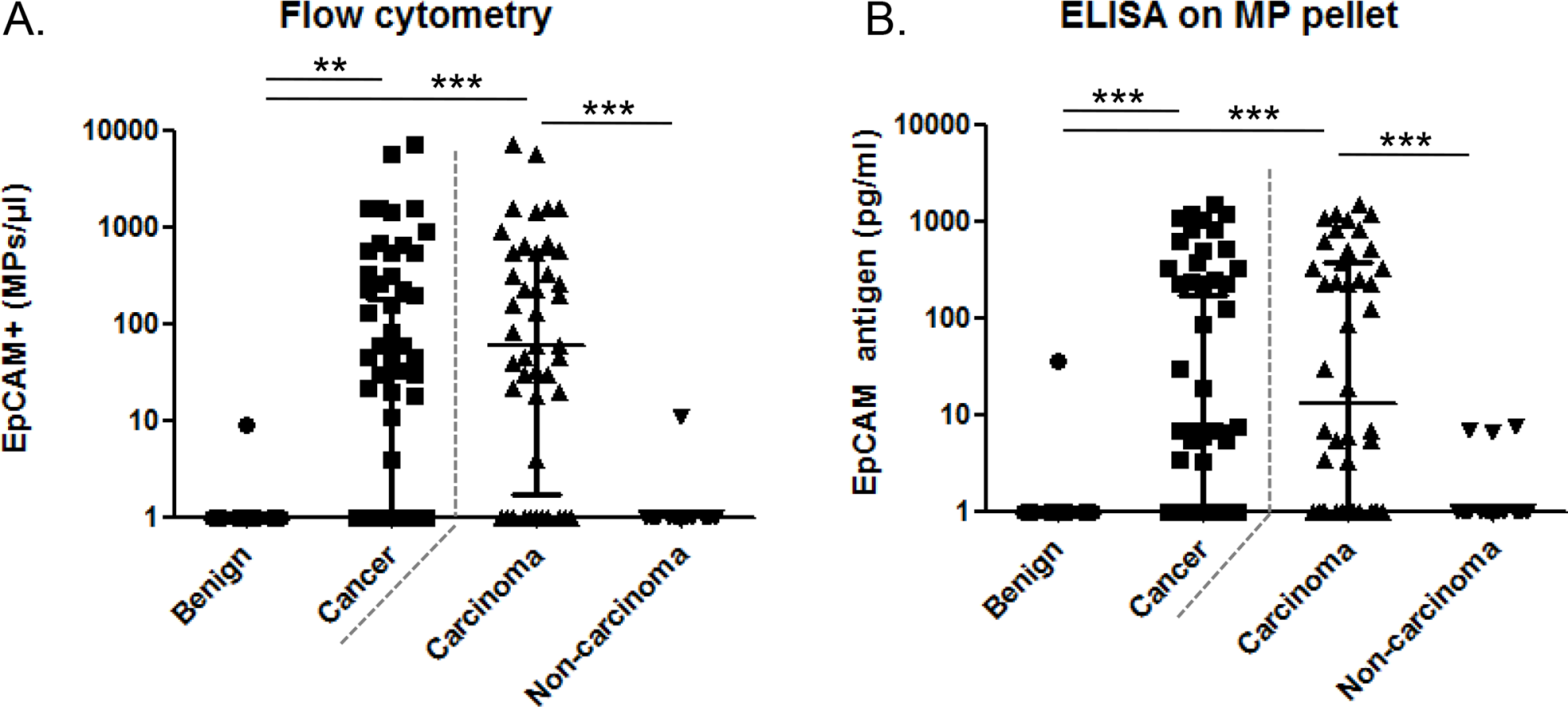
EpCAM+ microparticles in the pleural effusion according to the patient etiology **A.** AnnV+/EpCAM+ MPs enumeration by flow cytometry on benign (*n* = 14), cancerous (*n* = 71), carcinoma (*n* = 44) and non-carcinoma neoplasia (*n* = 27) in pleural fluids. **B.** AnnV+/EpCAM+ MPs enumeration by ELISA on purified MP pellets. ADK, adenocarcinoma; EpCAM, epithelial cell adhesion molecule.

Then, the capacity of EpCAM+ MPs to distinguish benign and MPE was evaluated by two methods : 1) high sensitive flow cytometry, performed directly on the pleural fluid and 2) ELISA for EpCAM antigen performed on the MP pellet after high speed centrifugation of the pleural fluid. As shown on Figure 3A, detection of EpCAM+ MPs by flow cytometry was found in 50% (35/70) of cancer patients. In these patients, the median level was 176 [37–665] EpCAM+ MPs/μl, median [25th–75th interquartile range]. Only 1 out of 14 patients with benign pleurisies presented a very low number of EpCAM+ MPs (9 MPs/μl). Using an ELISA test with a detection limit at 1 pg/ml, EpCAM+ MPs were found in 44% (32/71) of cancer patients with a median value of 228 [7–595] pg/ml (Figure 3B). Only 1 out of 14 patients with benign pleural effusions was positive. Thus, in this cohort of patient, the detection of EpCAM+ MPs showed a good specificity (sp = 93% for flow cytometry and ELISA) and a low sensitivity (Se = 49% and 45% for flow cytometry and ELISA, respectively) to distinguish between benign and MPE.

Because, EpCAM antigen is not expressed by all tumors, we divided malignant patients into two groups according to their etiology: 1) carcinoma (*n* = 44) known to generally express EpCAM, and 2) non-carcinoma (*n* = 27, mesothelioma, melanoma, lymphoma and sarcoma) generally not expressing EpCAM. By flow cytometry, EpCAM+ MPs were detected in 79% (34/43) of carcinoma pleurisies with 221 [42–670] EpCAM+ MPs/μl compared to only 1 out of 27 patients with non-carcinoma malignant pleurisies with a very low number of EpCAM+ MPs (11 MPs/μl) (Figure 3A). In contrast, using ELISA test, the detection of EpCAM+ MPs was positive in 66% (29/44) of carcinoma pleurisies with 233 [42–713] pg/ml but also in 3/27 with non-carcinoma malignant pleural effusions (mesothelioma) with a low concentration EpCAM (7 pg/ml) (Figure 3B). Thus, the detection of EpCAM+ MPs by flow cytometry showed a better specificity and sensitivity than by ELISA (Sp: 96% *vs* 89%; Se: 79% *vs* 66%) to distinguish between carcinoma and non-carcinoma MPE. Consequently flow cytometry was selected to measure EpCAM+ MPs in the next part of the study.

Among patients diagnosed as carcinoma, we therefore compared tumor cell detection by cytology (the reference method) to EpCAM+ MPs enumeration by flow cytometry, for their capacity of to differentiate benign and MPE (Table 1). Patients were stratified according to the presence of EpCAM+ MPs. A perfect agreement between tumoral cells and MPs was found for 68% of patients.

**Table 1:** EpCAM-positive MP detection and cytology in pleural effusions from primitive carcinoma

**DIAGNOSIS**	**EpCAM + MPs (FCM)**	**Malignant cells (Cytology)**
Lung ADK	+++	++
Lung ADK	+++	+++
Pancreas ADK	+++	+++
Lung ADK	+++	++
Lung ADK	+++	++
Ovarian ADK	+++	+
Breast ADK	+++	++
Lung ADK	+++	+++
Ovarian ADK	+++	+++
Lung ADK	+++	++
Thyroide ADK	+++	++
Lung ADK	+++	+
Lung ADK	+++	+
Breast ADK	+++	++
Neuroendocrin carcinoma	+++	+++
Undifferentiated carcinoma	+++	++
Lung ADK	+++	++
Lung ADK	+++	++
Lung ADK	++	++
Lung ADK	++	+
Lung ADK	++	+
Undifferentiated carcinoma	++	++
Prostate ADK	++	+
Squamous cell carcinoma	++	+
Lung ADK	+	+
Lung ADK	+	++
Lung ADK	+	+
Lung ADK	+	+
Lung ADK	+	+
Lung ADK	+	+
Prostate ADK	+++	−
Head and neck ADK	+	−
Breast ADK	+	−
Lung ADK	−	+
Prostate ADK	−	+++
Colon ADK	−	+++
Breast ADK	−	+
Squamous cell carcinoma	−	+
Pancreas ADK	−	−
Lung ADK	−	−
Lung ADK	−	−
Ovarian ADK	−	−
Lung ADK	−	−

ADK, adenocarcinoma; EpCAM, epithelial cell adhesion molecule; MPs, microparticles; ND, not determined. Positivity was determined by 1) flow cytometry (FCM) according to the following criteria: negative (−), < 2 MPs/μl; positive (+), 2–50 MPs/μl; (++), 50–100 MPs/μl; and (+++), > 100 MPs/μl. 2) Cytology : negative (−), absence; positive (+), < 5%; (++), 5–50%; and (+++), > 50% of malignant cells, respectively. Grey zones are no more visible in the table. discordant results between EpCAM + MPs and cytology

Interestingly, in 3 cases, only EpCAM+ MPs were detected. Conversely, cytology alone was positive in 5 cases for which, 3 pleural fluids presented less than 5% of malignant cells (+) and 2 more than 50% of malignant cells (+++). Notably, in these last two cases, the expression of EpCAM measured by immunocytochemistry was either negative or weak. Therefore, compared to cytology alone, combining cell detection by cytology and EpCAM+ MPs enumeration by flow cytometry improved the diagnosis of MPE (from 81% to 88%).

## DISCUSSION

Microvesiculation is a general process wich occurs at the membrane of virtually all cell types. Thus, MPs are theoretically present in all body fluids. Accordingly, they have been reported not only in peripheral blood (serum or plasma) but also in others biological fluids such as urine, cerebrospinal fluid, saliva, synovial and vitreous fluids [24]. However, pleural effusions, have been underexplored. Indeed, only the presence of exosomes purified by sequential ultracentrifugations on sucrose or iodixanol density gradient have been described in very small series of patients (3, 9 and 12 patients, respectively [9–11]). In the present study, we report for the first time the presence high amounts of MPs originating from normal and malignant cells in pleural fluids of 85 patients, opening the way for a potential non invasive biomarker for pleural diseases. Moreover, this study is the first description of tumor-derived MPs expressing the EpCAM antigen in the pleural liquid from lung carcinoma patients. Interestingly, the detection of EpCAM+ MPs by flow cytometry showed a better specificity and sensitivity than ELISA to distinguish between pleural carcinoma and the others malignant pleural effusions. Finally, combining flow cytometric enumeration of EpCAM+ MPs and cytology improved the diagnosis of MPE compared to cytology alone. This study establishes the basis for using EpCAM+ MPs as a promising biomarker that could be add to the armamentarium to distinguish benign and MPE.

EVs offer several benefits over current clinical biomarkers for cancer screening and diagnosis. They shuttle clinically validated biomarkers but they also represents a novel source of proteins and nucleic acids that could be exploited as surrogate biomarkers. Moreover, EVs protect their cargo from the attack of nucleases and proteases, increasing biomarker half-life [25]. In the litterature, most of the study demonstrating the biomarker potential of EVs associated with tumor antigen have focused on exosomes [26]. In patients with ovarian cancer, Taylor and colleagues identified a tumor-specific signature of eight miRNAs in EpCAM+ exosomes detectable in patient's plasma, as a disease-specific signature that discriminate cancer from benign ovarian disease [25]. A retrospective study in stage III and IV melanoma patients showed increased levels of caveolin-1- EVs in plasma with a sensitivity of 69% and specificity of 96.3% while levels of serum LDH were altered only in 12.5% of patients [27]. Serum prostate-specific antigen (PSA) has been found on plasma- and urine-derived exosomes in prostate cancer [28, 29]. In another report, exosomal survivin was identified as promising surrogate biomarker for early diagnosis of prostate cancers [30]. Plasma levels of survivin-positive-EVs were higher in prostate cancer patients than benign hyperplastic patients and healthy donors, potentially providing an alternative tool to reduce the number of false positives generated by the PSA test. The tumour-specific EGFRvIII was detected in serum EVs from glioblastoma patients which proved to be useful for monitor patient therapy [31, 32]. Taken together, most of these studies supporting the clinical potential of EVs as biomarkers for screening and early diagnosis of cancer have focused on exosomes, which detection is based on time-consumming assays such as western-blot or methods with limited availability such as micro-NMR [32]. In the present study, we identified MPs as valuable markers which have the advantage to be directly accessible by flow cytometry [15], a methodology largely available in diagnostic labs and providing a result in less than one hour. In addition, compared to plasma, the low background noise of the pleural fluid samples is an optimal preanalytic condition for MP measurement. In the present study, we showed that detection of tumoral-MPs by flow cytometry showed a better sensitivity than by ELISA to distinguish between pleural carcinoma and the others MPE. This difference may rely on the pre-analytical steps which differs between flow cytometry and ELISA; the later method includes a high speed centrifugation known to impact on the recovery of MPs whereas flow cytometry is directly performed on the native sample. Although the sensitivity and specificity of a diagnosis by tumoral MPs should be established in larger multicenter cohorts, this study establishes the basis for the detection of tumoral MPs by flow cytometry as potential biomarkers for the classification of pleural effusions.

Cytology is the gold standard method for the diagnosis of pleural effusion but presents limitations that rely on cell scarceness or difficulties to discriminate cancer cells from reactive mesothelial or inflammatory cells [33]. Thus, additional methods have been evaluated to improve the diagnostic accuracy and to avoid invasive diagnostic techniques, such as thoracoscopy [34]. Notably, a combination of cytology and RT-PCR analysis of CEA and Ep-CAM significantly improved the detection sensitivity of tumor cells in serous effusions [35]. In the present study, we showed that combining flow cytometric enumeration of EpCAM+ MPs and cytology improved the diagnosis of MPE compared to cytology alone. Detection of tumoral-MPs by flow cytometry offers also several advantages compared to cytology: 1) tumor-derived MPs are detectable despite a low number of tumoral cells due to the high amounts of MPs produced by tumor cells; 2) tumor-derived MPs are still detectable when the apoptotic parental cells are no longer detectable; therefore, MPs remain detectable in the pleural fluids after the cell degradation which is of particular interest in daily practice for old samples; 3) Detection of tumoral-MPs by flow cytometry is operator-independent and do not necessitate a cytological expertise. Beside allowing to mini-invasively discriminate benign and malignant pleural effusion, the detection of EpCAM+ MPs could be useful to monitor patient's treatment. Indeed, identification of patient with positive MPs for EpCAM allows to elicit candidate which may benefit from anti-EpCAM biotherapy such as catumaxomab [36, 37].

Because immunohistological detection of EpCAM in pleural fluid is usually difficult in case of low percentage of malignant cells, detection of MPs for EpCAM by flow cytometry may represent an advantageous companion test for personalized medicine targeting EpCAM. This biomarker could also be useful for mini-invasive monitoring of the patients during a specific treatment. In fact, patients with important comorbidities, poor performance status, or advanced age cannot easily undergo thoracoscopy. For the patient, a mini-invasive thoracentesis or the use of indwelling pleural catheter to remove pleural fluid could provide material suitable for a detailed analysis of the microparticles to obtain additional information about the status of the disease.

A limitation of this work is that the detection of tumoral-MPs was restricted to EpCAM+ events. EpCAM antigen is not expressed by all tumors, even not all carcinoma [38] which may explains false negative results for tumoral-MPs detection despite the presence of tumoral cells by cytology. Consistent with this hypothesis, in the two patients negative for EpCAM+ MPs and positive by cytology, the expression of EpCAM measured by immunocytochemistry was either negative or weak despite a large number of malignant cells. So we can assume that detection of malignancy markers on the surface of MPs could be enlarged to the detection of others tumoral antigens such as PSA, EGFR-vIII, CD24 or mesothelin.

This work demonstrating the presence of tumoral MPs expressing EpCAM in pleural fluids also opens new directions about the MPs role in cancer progression. Indeed, in patients with gliomas, it has been reported that EpCAM overexpression correlates significantly with malignancy (WHO grades), proliferation (Ki67), angiogenesis, and prognosis [39]. On the other hand, MPs vectorize nucleic acid molecules such as miRNAs, miRNAs, ncRNAs, DNA, cDNA and retrotransposons originating from parent cells. The recent discovery of EpCAM involvement in cell signaling and breast cancer invasion suggests that its vectorization by MPs could contribute to carcinogenesis [40]. Since tumoral MPs also carry various features (including procoagulant, proteolytic or inflammatory activities) and genetic signatures implicated in malignant responses, it can be assumed that EpCAM+ MPs could behave as relevant players of carcinogenesis in the pleural microenvironment.

## PATIENTS AND METHODS

### Patients

Eighty-five patients (30 females and 55 males) with pleural effusions were prospectively included in this study. Among them, 71 consecutive patients with histologically-proven primary or metastatic pleural cancer were included. There were 44 patients with carcinoma and 27 patients with others pleural cancers (16 mesotheliomas, 4 melanomas, 3 lymphomas, 2 breast cancers, 1 uterine sarcoma, 1 ovarian cancer). In addition to these patients diagnosed with malignant pleurisy, we enrolled 14 patients with cytologically negative pleural effusions without neoplastic or atypical cells. These patients were not significantly different in gender and age with cancer patients. Informed consent was obtained from the patients or their relatives, and the study was approved by the institutional ethical committee according to local regulation. The cytological diagnosis was performed by an expert cytopathologist, and the findings were reported as “cytologically-positive” in case of significant atypical cells or malignant cells in the pleura and “cytologically-negative” in the other cases [13]. Thoracoscopy was standardized in accordance to current European practice and used a standardized telescope (R. Wolf GmbH, Knittlingen, Germany) to obtain multiple parietal pleura biopsies for the histological diagnosis [14].

### Sample processing

The pleural fluids were centrifuged at 300 g for 10 min and at 1,000 g for 15 min at room temperature with light braking in order to remove cells and avoid artifactual generation of MPs. The collected supernatant was stored at −80°C until use. For ELISA, the MPs were purified by high-speed centrifugation at 70,000 g for 90 min at 4°C as already published [15]. The pelleted MPs were washed twice (70,000 g for 90 min at 4°C) and re-suspended in phosphate-buffered saline (PBS). The supernatant from the last washing was used as the negative control.

### MP enumeration by flow cytometry

The MPs were enumerated by highly sensitive flow cytometry, as previously described [16]. Briefly, 30 μL of pleural fluid were incubated with the appropriate amount of specific antibodies and 10 μL of AnnV-FITC (fluorescein, Beckman-Coulter, Miami, FL, USA). A fluorescent-matched irrelevant antibody was used as a control for the specific antibodies. Each stained sample was analyzed on a NAVIOS 3-laser instrument (Beckman-Coulter), following a protocol standardized with Megamix-Plus FSC beads (BioCytex, Marseille, France). The platelet-, erythrocyte-, leukocyte- and endothelial-derived MPs were defined as AnnV+/CD41+, AnnV+/CD235a+, AnnV+/CD11b+ or AnnV+/CD31+/CD41−, respectively. Positivity for podoplanin (PE conjugated, phycoerythrin; Biolegend, San Diego, USA), EpCAM (PE; clone EBA-1, BD Bioscience, San Jose, USA), and mucin 1 (FITC conjugated; eBioscience, San Diego, USA) was determined by the AnnV+ events. The absolute MP counts (events per μL) were determined using *ad hoc* counting beads (CytoCount®, Dako, Copenhagen, Denmark).

To verify the specificity of the EpCAM signal using flow cytometry, the EpCAM+ MPs were depleted by immunomagnetic separation using beads (8 × 10^8^ beads/mL; Dynabeads, Invitrogen, Oslo, Norway) coated with purified EpCAM (clone KS1/4; BD Biosciences). The control experiments were performed in parallel using beads coated with a non-specific antibody (IgG1, 8 × 10^8^ beads/mL; Dynabeads, Invitrogen).

### Cryo-transmission electron microscopy (TEM)

For the cryo-TEM experiments, 4 μL of pleural liquid was deposited on an electron microscopy (EM) grid coated with a perforated carbon film (Ted Pella, USA). The excess liquid was blotted off with filter paper, and the grid was quickly plunged into liquid ethane in a Leica EM-CPC cryo-chamber. The EM grids were stored in cryo-boxes under liquid nitrogen until use. For the cryo-TEM observations, the EM grids were mounted in a Gatan 626 cryo-holder and transferred into a Tecnai F20 microscope operated at 200 kV. The images were recorded with a USC1000-SSCCD Gatan camera.

### Measurement of EpCAM-positive MPs using ELISA

EpCAM was measured by a DuoSet ELISA (enzyme linked immunoSorbent assay) kit (R&D Systems, Minneapolis, MN, USA) in the pleural fluids or purified MPs according to the manufacturer's instructions. Mouse anti-human EpCAM, at a concentration of 4 μg/mL in phosphate-buffered saline (PBS), was coated on 96-well Costar EIA plates and incubated overnight at room temperature. Then, 100 μL of pleural fluid, purified MPs or corresponding last-wash supernatant were incubated at room temperature for 2 h. Following the washing steps, EpCAM was detected with a biotinylated/peroxidase-streptavidin goat anti-human EpCAM antibody (200 ng/mL). The concentration was determined by measuring the optical density at 450 nm. According to the sensitivity of this test, results > 2 pg/mL were considered positive.

### Statistical analysis

The statistical analyses were performed with GraphPad Prism software v.5.0 (GraphPad Software, San Diego, CA, USA). The continuous variables are reported as medians and the 25–75th interquartile ranges. A Mann-Whitney test was used for the quantitative variables. The reported *p*-values are 2-sided, and *p* < 0.05 was considered significant.

## References

[1] Antony VB, Loddenkemper R, Astoul P, Boutin C, Goldstraw P, Hott J, Rodriguez Panadero F, Sahn SA (2001). Management of malignant pleural effusions. Eur Respir J.

[2] Rodriguez-Panadero F, Borderas Naranjo F, Lopez Mejias J (1989). Pleural metastatic tumours and effusions. Frequency and pathogenic mechanisms in a post-mortem series. Eur Respir J.

[3] Light RW (2011). Pleural effusions. Med Clin North Am.

[4] Freyssinet JM (2003). Cellular microparticles: what are they bad or good for?. J Thromb Haemost.

[5] Burnier L, Fontana P, Kwak BR, Angelillo-Scherrer A (2009). Cell-derived microparticles in haemostasis and vascular medicine. Thromb Haemost.

[6] De Broe ME, Wieme RJ, Logghe GN, Roels F (1977). Spontaneous shedding of plasma membrane fragments by human cells *in vivo* and *in vitro*. Clin Chim Acta.

[7] Berckmans RJ, Nieuwland R, Tak PP, Boing AN, Romijn FP, Kraan MC, Breedveld FC, Hack CE, Sturk A (2002). Cell-derived microparticles in synovial fluid from inflamed arthritic joints support coagulation exclusively via a factor VII-dependent mechanism. Arthritis Rheum.

[8] van Blitterswijk WJ, Emmelot P, Hilkmann HA, Hilgers J, Feltkamp CA (1979). Rigid plasma-membrane-derived vesicles, enriched in tumour-associated surface antigens (MLr), occurring in the ascites fluid of a murine leukaemia (GRSL). Int J Cancer.

[9] Rupp AK, Rupp C, Keller S, Brase JC, Ehehalt R, Fogel M, Moldenhauer G, Marme F, Sultmann H, Altevogt P (2011). Loss of EpCAM expression in breast cancer derived serum exosomes: role of proteolytic cleavage. Gynecol Oncol.

[10] Bard MP, Hegmans JP, Hemmes A, Luider TM, Willemsen R, Severijnen LA, van Meerbeeck JP, Burgers SA, Hoogsteden HC, Lambrecht BN (2004). Proteomic analysis of exosomes isolated from human malignant pleural effusions. Am J Respir Cell Mol Biol.

[11] Park JO, Choi DY, Choi DS, Kim HJ, Kang JW, Jung JH, Lee JH, Kim J, Freeman MR, Lee KY, Gho YS, Kim KP (2013). Identification and characterization of proteins isolated from microvesicles derived from human lung cancer pleural effusions. Proteomics.

[12] D'Souza-Schorey C, Clancy JW (2012). Tumor-derived microvesicles: shedding light on novel microenvironment modulators and prospective cancer biomarkers. Genes Dev.

[13] Whitaker D (2000). The cytology of malignant mesothelioma. Cytopathology.

[14] Rodriguez-Panadero F, Janssen JP, Astoul P (2006). Thoracoscopy: general overview and place in the diagnosis and management of pleural effusion. Eur Respir J.

[15] Berda-Haddad Y, Robert S, Salers P, Zekraoui L, Farnarier C, Dinarello CA, Dignat-George F, Kaplanski G (2011). Sterile inflammation of endothelial cell-derived apoptotic bodies is mediated by interleukin-1alpha. Proc Natl Acad Sci U S A.

[16] Robert S, Lacroix R, Poncelet P, Harhouri K, Bouriche T, Judicone C, Wischhusen J, Arnaud L, Dignat-George F (2012). High-sensitivity flow cytometry provides access to standardized measurement of small-size microparticles—brief report. Arterioscler Thromb Vasc Biol.

[17] Hjerpe A, Ascoli V, Bedrossian CW, Boon ME, Creaney J, Davidson B, Dejmek A, Dobra K, Fassina A, Field A, Firat P, Kamei T, Kobayashi T (2015). Guidelines for the cytopathologic diagnosis of epithelioid and mixed-type malignant mesothelioma: Complementary Statement from the International Mesothelioma Interest Group, Also Endorsed by the International Academy of Cytology and the Papanicolaou Society of Cytopathology. Diagn Cytopathol.

[18] Kono T, Shimoda M, Takahashi M, Matsumoto K, Yoshimoto T, Mizutani M, Tabata C, Okoshi K, Wada H, Kubo H (2007). Immunohistochemical detection of the lymphatic marker podoplanin in diverse types of human cancer cells using a novel antibody. Int J Oncol.

[19] Raica M, Cimpean AM, Ribatti D (2008). The role of podoplanin in tumor progression and metastasis. Anticancer Res.

[20] Breiteneder-Geleff S, Soleiman A, Kowalski H, Horvat R, Amann G, Kriehuber E, Diem K, Weninger W, Tschachler E, Alitalo K, Kerjaschki D (1999). Angiosarcomas express mixed endothelial phenotypes of blood and lymphatic capillaries: podoplanin as a specific marker for lymphatic endothelium. Am J Pathol.

[21] Saharinen P, Tammela T, Karkkainen MJ, Alitalo K (2004). Lymphatic vasculature: development, molecular regulation and role in tumor metastasis and inflammation. Trends Immunol.

[22] Nath S, Mukherjee P (2014). MUC1: a multifaceted oncoprotein with a key role in cancer progression. Trends Mol Med.

[23] Lau SK, Weiss LM, Chu PG (2004). Differential expression of MUC1, MUC2, and MUC5AC in carcinomas of various sites: an immunohistochemical study. Am J Clin Pathol.

[24] Piccin A, Murphy WG, Smith OP (2007). Circulating microparticles: pathophysiology and clinical implications. Blood Rev.

[25] Taylor DD, Gercel-Taylor C (2008). MicroRNA signatures of tumor-derived exosomes as diagnostic biomarkers of ovarian cancer. Gynecol Oncol.

[26] Zocco D, Ferruzzi P, Cappello F, Kuo WP, Fais S (2014). Extracellular vesicles as shuttles of tumor biomarkers and anti-tumor drugs. Front Oncol.

[27] Logozzi M, De Milito A, Lugini L, Borghi M, Calabro L, Spada M, Perdicchio M, Marino ML, Federici C, Iessi E, Brambilla D, Venturi G, Lozupone F (2009). High levels of exosomes expressing CD63 and caveolin-1 in plasma of melanoma patients. PLoS One.

[28] Mizutani K, Terazawa R, Kameyama K, Kato T, Horie K, Tsuchiya T, Seike K, Ehara H, Fujita Y, Kawakami K, Ito M, Deguchi T (2014). Isolation of prostate cancer-related exosomes. Anticancer Res.

[29] Mitchell PJ, Welton J, Staffurth J, Court J, Mason MD, Tabi Z, Clayton A (2009). Can urinary exosomes act as treatment response markers in prostate cancer?. J Transl Med.

[30] Khan S, Jutzy JM, Valenzuela MM, Turay D, Aspe JR, Ashok A, Mirshahidi S, Mercola D, Lilly MB, Wall NR (2012). Plasma-derived exosomal survivin, a plausible biomarker for early detection of prostate cancer. PLoS One.

[31] Skog J, Wurdinger T, van Rijn S, Meijer DH, Gainche L, Sena-Esteves M, Curry WT, Carter BS, Krichevsky AM, Breakefield XO (2008). Glioblastoma microvesicles transport RNA and proteins that promote tumour growth and provide diagnostic biomarkers. Nat Cell Biol.

[32] Shao H, Chung J, Balaj L, Charest A, Bigner DD, Carter BS, Hochberg FH, Breakefield XO, Weissleder R, Lee H (2012). Protein typing of circulating microvesicles allows real-time monitoring of glioblastoma therapy. Nat Med.

[33] Bedrossian CW (1998). Diagnostic problems in serous effusions. Diagn Cytopathol.

[34] Mohanty SK, Dey P (2003). Serous effusions: diagnosis of malignancy beyond cytomorphology. An analytic review. Postgrad Med J.

[35] Passebosc-Faure K, Li G, Lambert C, Cottier M, Gentil-Perret A, Fournel P, Perol M, Genin C (2005). Evaluation of a panel of molecular markers for the diagnosis of malignant serous effusions. Clin Cancer Res.

[36] Bokemeyer C (2010). Catumaxomab—trifunctional anti-EpCAM antibody used to treat malignant ascites. Expert Opin Biol Ther.

[37] Berek JS, Edwards RP, Parker LP, DeMars LR, Herzog TJ, Lentz SS, Morris RT, Akerley WL, Holloway RW, Method MW, Plaxe SC, Walker JL, Friccius-Quecke H (2014). Catumaxomab for the treatment of malignant ascites in patients with chemotherapy-refractory ovarian cancer: a phase II study. Int J Gynecol Cancer.

[38] Patriarca C, Macchi RM, Marschner AK, Mellstedt H (2012). Epithelial cell adhesion molecule expression (CD326) in cancer: a short review. Cancer Treat Rev.

[39] Chen X, Ma WY, Xu SC, Liang Y, Fu YB, Pang B, Xin T, Fan HT, Zhang R, Luo JG, Kang WQ, Wang M, Pang Q (2014). The overexpression of epithelial cell adhesion molecule (EpCAM) in glioma. J Neurooncol.

[40] Sankpal NV, Fleming TP, Gillanders WE (2013). EpCAM modulates NF-kappaB signaling and interleukin-8 expression in breast cancer. Mol Cancer Res.

